# The Relationship between Job Demands and Turnover Intention among Chinese Prison Officers during the COVID-19 Pandemic: A Moderated Mediation Model

**DOI:** 10.3390/bs13070558

**Published:** 2023-07-05

**Authors:** Yuze Zeng, Qingqi Zhang, Junze Xiao, Ke Qi, Ai Ma, Xiaoqian Liu

**Affiliations:** 1 School of Criminal Justice, China University of Political Science and Law, Beijing 102249, China; 2 School of Sociology, China University of Political Science and Law, Beijing 102249, China; 3 The Psychological Counseling Center, China University of Political Science and Law, Beijing 102249, China

**Keywords:** prison officer, COVID-19, job demands, turnover intention, burnout, perceived efficacy in overcoming COVID-19 pandemic

## Abstract

The COVID-19 pandemic has brought enormous challenges to both employees and organizations all over the world. Previous studies have found high turnover rates among prison officers since the outbreak of COVID-19. This cross-sectional study aimed to investigate the mediating role of job burnout between job demands and turnover intention, as well as the moderating role of the perceived efficacy in overcoming COVID-19 in Chinese prison officers. In total, 1316 prison officers were recruited to complete an online questionnaire between May 2022 and June 2022 (during the COVID-19 pandemic). The bootstrapping approach was used to assess the moderated mediation model in this study. The results showed that prison officers’ job demands were positively associated with their turnover intention. Job burnout mediated the relationship between job demands and turnover intention. Perceived efficacy in overcoming COVID-19 moderated the effect of job burnout on turnover intention. Based on these results, suggestions were provided to reduce the high turnover rate of prison officers in public health events like the COVID-19 pandemic.

## 1. Introduction

During the novel coronavirus pneumonia (COVID-19) pandemic, overlooked professions have faced daunting challenges. Prison officers, as our ‘urban heroes’, were confronted with unprecedented pressures, with the pandemic making their working conditions more arduous. In December 2019, COVID-19 was identified as a new acute infectious disease with a high incidence and infectivity rate. According to the WHO data, globally, as of 21 June 2023, more than 768 million individuals have contracted the COVID-19 virus, and more than 6.9 million have died, respectively. This pandemic plunged the world into a public crisis involving politics, education, economy, society, environment, and all aspects of life [1]. As a worldwide epidemic, the COVID-19 outbreak, viewed as “a humanitarian crisis” [2], has caused an increased workload on pandemic prevention for most jobs. Recent studies have demonstrated the adverse effect of the spread of COVID-19 on staff’s performances, including procrastination, go-slow, and even turnover [3]. For instance, Abdalla [4] found that employee intentions to leave increased due to the COVID-19 crisis. It is of great significance for managers to consider how to retain qualified employees during the pandemic.

The job of a prison officer has gradually become a laborious occupation with a high turnover rate [5]. Different from other professionals, prison officers are more likely to feel anxious and burned out due to remote workplaces, intensive work, irregular attendance times, and isolated working environments. Instead of telecommuting like other professions, during the COVID-19 pandemic, prison officers had to stay in prison for a long time and with increased job demands, which made them face more pressure than other workers during the pandemic. Prison officers should not only pay attention to their physical health, but also need to take protective measures for prisoners. In addition to daily checks (e.g., nucleic acid tests and temperature checks), prison officers have to develop and carry out pandemic prevention plans, conduct statistical work frequently, and strengthen management in prison; prison officers are worried about the widespread of COVID-19 in prisons caused by either lax supervision or their mistakes, which thereby increases their anxiety and burnout invisibly. Thus, the COVID-19 pandemic has posed unexpected difficulties and incremental job demands (both workload and emotional demands) for this group. Prison officers are among the frontline workers at a high risk of exposure to COVID-19. Evaluating the association between job demands and quit intention among prison officers during the COVID-19 pandemic is essential, which can help formulate targeted interventions to support prison officers in the COVID-19 context and reduce turnover rates.

Notably, though the COVID-19 pandemic has subsided, and COVID-19 prevention has become commonplace, future infectious pandemics are likely to emerge. Conducting research in the context of the COVID-19 pandemic has important implications for the future. This study was designed to investigate the effects of job demands on turnover intention and the underlying mechanisms among prison officers during the COVID-19 pandemic, taking into account job burnout as a mediating variable and the perceived efficacy in overcoming COVID-19 as a moderating variable in order to provide recommendations for reducing the high turnover rate of prisons in similar situations in the future.

### 1.1. Job Demands and Turnover Intention

Job demands are defined as the physical, mental, social, or organizational parts of a job that require continued physical and/or mental efforts and, as a result, come with some physical and/or mental costs [6]. Consistent with this definition, prison officers’ job demands during the COVID-19 pandemic consisted of workload (the amount of work that had to be carried out) and emotional demands (emotional labor needed for work). The term “turnover intention” refers to an employee’s perception of the likelihood that they will leave their current position or company due to a variety of factors [7]. A meta-analysis encompassing multiple work contexts and employee populations showed that high job demands are associated with higher turnover intentions [8], implying that when employees face a greater workload or emotional labor, they are more likely to consider leaving.

In reality, prison officers are responsible for maintaining order and safety within correctional facilities. Their work is both physically and emotionally demanding and requires them to work long hours in a high-stress environment. The COVID-19 pandemic has further exacerbated the particularity of correctional officers’ work. The need to maintain social distancing and other safety protocols has increased the workload [9] of those who are responsible for enforcing these measures. Throughout the COVID-19 pandemic, prison officers have had to work faster, conduct multiple tasks simultaneously, and take fewer breaks, which may adversely render high stress, and result in a higher turnover intention [10].

### 1.2. Job Burnout as a Mediator

The definition of burnout focuses on the mental, emotional, and physical tiredness brought on by excessive job demands [11]. Maslash [12] indicated emotional exhaustion (the sensation of being emotionally depleted), cynicism (pessimistic viewpoints toward clients), and lowered self-worth (poor assessment of own efforts and achievements) as three elements of job burnout. Burnout is a common phenomenon in the workplace, resulting from the accumulation of heavy job demands [13].

The job demands–resources (JD–R) theory claims that job demands are linked to burnout [6]. The energy-draining demands of a job contribute to increased fatigue. The JD–R model asserts that exhaustion levels tend to rise with the increased degree of job demands [14]. Consistent with the JD–R model, a study carried out among Norwegian police officers found that the cumulative impact of high work demands, and inadequate autonomy led to exhaustion [15]. Since the widespread of the COVID-19 pandemic, prevention and statistic work have become the daily routines for prison officers, increasing their workload as a consequence. High-intensity work for a long time may consume individuals’ physical and psychological resources, eventually leading to job burnout [16].

To prevent the COVID-19 from spreading wide, prison officers need to maintain their concentration and vigilance at work [17], which may further lead to burnout as a result. Over time, prison officers may work less efficiently, and become pessimistic about the future, leading to a strong desire to resign. Previous research has revealed that job burnout is a significant factor affecting turnover intention. This conclusion has been validated not only in different occupational groups, such as nurses, psychologists, and workshop workers in manufacturing companies [18,19,20], but also across different generations [21]. In addition, when understanding the reasons for turnover intention, the Hom-Griffeth‘s employee turnover model provides a powerful framework. This model views turnover as a multi-stage process, starting with the job demands [22]. Within the model, job demands, such as an excessive work pressure or workload can decrease satisfaction and trigger job burnout [23], which may ultimately lead to turnover intention [24].

Thus, job demands, which have increased significantly during the COVID-19 pandemic, could positively predict job burnout in prison officers. Furthermore, prison officers with high job burnout lose enthusiasm for their work and feel hopeless, anxious, and stressed, resulting in a strong intention to leave. It is therefore likely that job demands can influence turnover intentions through job burnout.

### 1.3. Perceived Efficacy in Overcoming the COVID-19 Pandemic as a Moderator

A perceived efficacy in overcoming COVID-19 is one’s confidence to overcome the pandemic and the perceived effectiveness of the coping strategies used for the COVID-19 pandemic [25]. Gabriele et al. [26] pointed out that this perceived efficacy is one of the individual factors affecting the attitudes toward COVID-19. The extended parallel process model (EPPM) states that when faced with adversity, individuals respond adaptively if they believe that they have the ability to overcome it (e.g., tell themselves that the difficulties are only temporary); otherwise, they are likely to react negatively (e.g., reinforce pessimistic beliefs about COVID-19) [27]. With increasing job demands and burnout, prison officers are not static observers, but are rather dynamic actors. Dai et al. [28] found that throughout the COVID-19 pandemic, perceived efficacy was crucial for controlling one’s unpleasant feelings. That is, this perceived efficacy may promote individuals to adopt more constructive approaches to confront adverse work events (e.g., increased job demands).

A previous empirical study conducted on employees of a Chinese franchise company found that self-efficacy moderated the relationship between job burnout and turnover intention. This means that when individuals believe that they can handle the various negative impacts of job burnout, they were thereby less likely to increase their turnover intention, even if they experience a high level of job burnout [29]. Meanwhile, during the pandemic, the increase of job burnout among Chinese prison police officers was linked to a series of work policy changes related to the COVID-19 pandemic [30]. We can speculate that those with a high efficacy in overcoming the COVID-19 pandemic would be very optimistic in that as the epidemic was controlled, the roots of high job burnout will also reduce as a result. Thus, they would not contemplate leaving their jobs due to their current job burnout. That is, the perceived efficacy in overcoming the COVID-19 pandemic may play a moderating role in the relationship between job burnout and turnover intention. Specifically, when experiencing job burnout during the pandemic, compared with prison officers with a lower level of efficacy in overcoming the COVID-19 pandemic, those with a higher perceived efficacy were more likely to believe that the pandemic would eventually be defeated and that the current uncomfortable work situation would not last long, resulting in a lower level of turnover intention.

Does a perceived efficacy in overcoming COVID-19 moderate the influence of job demands on burnout among prison officers? The answer is probably no. The feeling of being able to overcome the pandemic may help individuals maintain a better professional attitude even in the face of a high burnout; however, it cannot alleviate the tremendous physical and psychological stress that high-demand jobs place on employees. Feeling burnout at work is a direct and immediate psychological response to high-intensity job demands. This is an automatic reaction, which is hard to be influenced through cognition or attitude.

### 1.4. The Current Study

The relationships among job demands, job burnout, and turnover intention have been validated across several different occupational groups, such as nurses, teachers, and corporate employees [31]. However, there has not been any related research conducted specifically within the context of the COVID-19 pandemic, and on the population of prison officers, who are an easily overlooked but very important occupational group. Furthermore, the COVID-19 pandemic has affected the work environment and the mental health of prison officers [32,33], which threatens the safety and stability of the prisons. Therefore, exploring the occupational psychological status of prison officers during the COVID-19 pandemic is of an utmost importance for maintaining their mental health, and reducing turnover rates in prisons under similar future circumstances.

**Figure 1:**
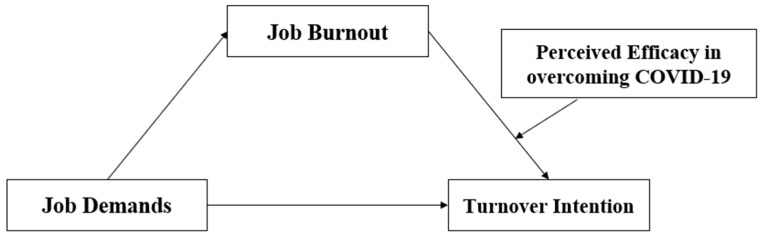
The proposed theoretical model.

To sum up, the present study investigated the association between job demands and turnover intention among prison officers by applying a hypothesized moderated mediation model that involves the roles of job burnout and perceived efficacy in overcoming the COVID-19 pandemic (Figure 1). Therefore, we proposed the following hypotheses:

**Hypothesis** **1** **(H1).** *Prison officers’ job demands will be linked to turnover intention positively during the COVID-19 pandemic.*

**Hypothesis** **2** **(H2).** *The relationship between job demands and turnover intention is mediated by burnout among prison officers.*

**Hypothesis** **3** **(H3).** *Perceived efficacy in overcoming the COVID-19 pandemic moderates the relationship between burnout and turnover intention.*

## 2. Methods

### 2.1. Participants and Procedure

This study was conducted in May and June of 2022. During the pandemic, it was difficult to fill in paper questionnaires face-to-face because of the completely enclosed management of prisons in China. This study collected data through a reliable online platform (www.wjx.cn (accessed on 18 May 2022)), which is the most widely used online survey platform in China promoting a high level of confidentiality. In total, 1459 prison officers from the Yunnan and Sichuan provinces completed the questionnaires. The criteria for participation in this study were as follows: being at least 20 years old, having worked in the prison system for at least one year, and having no history of mental disorders. In order to ensure the sample’s representativeness and generalizability, we collaborated with multiple prisons and employed stratified sampling techniques based on the prison’s size and location.

After reviewing the data for wrong, incomplete, and half-hearted responses, 143 responses were excluded. Finally, 1316 complete surveys were analyzed, representing a response rate of 90.20%.

To effectively control the possibility of bias, the following procedures were implemented: (a) all participants were informed that their answers would be anonymized for privacy considerations, and that the data would be used only for research purposes; (b) the order of the scales and items was randomized. The Ethics Committee of the School of Sociology at China University of Political Science and Law approved all research procedures.

### 2.2. Measures

#### 2.2.1. Job Demands

The demands subscale of the Copenhagen Psychosocial Questionnaire (COPSOQ) was established by Kristenson et al. [34] and amended by Meng et al. [35]. With a Cronbach’s alpha value of 0.70 for the majority of the scales, the Chinese version of this subscale was found to be reliable and valid when administered to a population of varying occupations [36,37]. This subscale consisted of two sections: workload (the amount of work that has to be carried out) and emotional demands (the emotional labor needed for work). It consisted of six items, each scored on a Likert scale ranging from 1 (strongly disagree) to 5 (strongly agree). A higher overall score indicated higher job demands. Item 3 required reverse scoring before the calculation of the total score. All the items in this scale were in the context of the COVID-19 pandemic. We added “during the pandemic of COVID-19” at the beginning or end of each item without any other changes. Sample items included “Do you have sufficient time to complete your job duties during the pandemic of COVID-19?”, and “During the pandemic of COVID-19, is it a requirement of your job that you conceal your emotions?”. In this study, the job demands subscale received an acceptable Cronbach’s alpha value of 0.72.

#### 2.2.2. Job Burnout

Job burnout was measured using the Maslach burnout inventory–general survey (MBI–GS) [38]. The Chinese version of this scale was revised by Li and Shi [39]. Fifteen items were measured across the three dimensions using a six-point scale. All the items on this scale were in the context of the COVID-19 pandemic. We added “during the pandemic of COVID-19” at the beginning or end of each item. The emotional exhaustion subscale consisted of five items, such as, “During the pandemic of COVID-19, I am always drained and dread the start of the workday”. The cynicism subscale included four items, for example, “During the pandemic of COVID-19, my enthusiasm for my job has decreased”, and “During the pandemic of COVID-19, I have become more cynical about the contribution of my job”. The reduced personal accomplishment subscale consisted of six items, such as “During the pandemic of COVID-19, I believe I’m contributing effectively to what this organization does”. Each item was scored on a Likert scale from 0 (never) to 6 (every day). Six positively worded items (items 10, 11, 12, 13, 14, and 15, respectively) required reverse scoring. Higher scores indicated higher levels of job burnout. The MBI–GS showed a Cronbach’s alpha value of 0.90 for the full scale (items 1–15), while the Cronbach’s alpha of the three dimensions ranged from 0.90 to 0.95, respectively.

#### 2.2.3. Turnover Intention

The turnover intention scale (TIS) was established by Mobley et al. [40] and was subsequently translated into Chinese by Huang [41]. Chinese prison officers have since confirmed its reliability and validity [42,43]. It included three items rated on a 5-point Likert scale from 1 (strongly disagree) to 5 (strongly agree). The higher the score, the higher the degree of turnover intention. These three items were modified in the context of the COVID-19 pandemic. We added “during the pandemic of COVID-19” at the beginning of them. For example, “During the epidemic of COVID-19, if there are other suitable job opportunities, I will accept them”. A Cronbach’s alpha value of TIS was obtained as 0.80 in this study.

#### 2.2.4. Perceived Efficacy in Overcoming the COVID-19 Pandemic

Perceived efficacy in overcoming the COVID-19 pandemic was measured with a well-established scale [44]. Both its reliability and validity have been verified by Dai et al. [28]. It included four items (e.g., “I think the pandemic will be completely under control in the near future.”) rated on a Likert scale from 1 (strongly disagree) to 7 (strongly agree). A higher score indicated a higher level of perceived efficacy in overcoming the COVID-19 pandemic. The Cronbach’s alpha for this scale was 0.85.

#### 2.2.5. Control Variables

The following variables were controlled to prevent their interference with the results: gender (1 = male, and 2 = female), age (1 = 20–29, 2 = 30–39, 3 = 40–49, and 4 = 50 and above), marital status (1 = single, 2 = married, 3 = divorced, and 4 = widowed), education (1 = high school, 2 = college, 3 = bachelor, and 4 = master or above) and income (1 = ≤¥50,000, 2 = ¥50,001–¥100,000, 3 = ¥100,001–¥150,000, 4 = ¥150,001–¥200,000, and 5 = >¥200,000), which were all included as covariates since previous studies have found significant associations between these demographic variables and turnover intention [45,46,47,48,49].

### 2.3. Data Analysis

We used SPSS 25.0 (IBM) for data analyses, including the common method deviation test, descriptive statistics, and Pearson’s correlation analysis. Furthermore, we assessed our hypotheses using the Hayes PROCESS macro [50]. The Hayes PROCESS macro allows researchers to examine the relationships of the variables and test the moderation effects. By employing the Hayes PROCESS macro with 5000 bootstrap samples and 95% confidence intervals, we obtained estimates of the moderation effects and their significance levels. Prior to conducting the analysis, all quantitative variables were standardized to ensure comparability and mitigate the potential impacts of the scale differences among the variables.

## 3. Results

### 3.1. Demographic Statistics

Demographic details of the participants are shown in Table 1. Of the 1316 respondents, 58.2% were male, and 41.8% were female, respectively. In terms of age distribution, approximately half (50.9%) were aged from 30 to 49 years old. Over four-fifths (82.1%) of the sample were married. With regard to education, the majority (77.7%) had a bachelor’s degree. Almost half (49.9%) earned ¥ 100,001–¥ 150,000 per year, while prison officers with an annual income of ¥ 50,001–¥ 100,000 accounted for 30.2%, respectively.

### 3.2. Common Method Deviation Test (CMV)

Common method variance (CMV) refers to the factors that can artificially inflate the relationships of the variables measured in the same study, leading to spurious associations. By conducting the CMV test, we aimed to assess the common method variance in our data. Harman’s single-factor test was performed to examine the CMV. The Kaiser–Meyer–Olkin value of unrotated factor analysis was 0.91, and the Bartlett’s test of sphericity yielded a statistically significant result (*p* < 0.001). The analysis yielded six factors, and the explanatory power of the first factor was 31.29%, which was less than the judgment standard of 40% [51], indicating that no CMV problem existed in this study.

### 3.3. Descriptive Statistics and Correlation Analysis

The results of the descriptive statistics and correlations among the measured variables are presented in Table 2. As hypothesized, job demands were found to be positively correlated with job burnout (*r* = 0.53, *p* < 0.01) and turnover intention (*r* = 0.32, *p* < 0.01), but were also found to be negatively correlated with the perceived efficacy in overcoming the COVID-19 pandemic (*r* = −0.25, *p* < 0.01). Job burnout was positively correlated with turnover intention (*r* = 0.28, *p* < 0.01), but was negatively correlated with perceived efficacy (*r* = −0.39, *p* < 0.01). Additionally, a weak negative correlation was found between turnover intention and perceived efficacy in overcoming the COVID-19 pandemic (*r* = −0.19, *p* < 0.01).

### 3.4. Mediation Analyses

All quantitative variables were standardized before analysis. Model 4 of the PROCESS macro [50] was used to assess the mediating effect of job burnout between job demands and turnover intention after controlling for gender, age, marital status, education, and income. As shown in Table 3, job demands positively predicted job burnout (*β* = 0.52, *SE* = 0.02, *p* < 0.001) and turnover intention (*β* = 0.15, *SE* = 0.03, *p* < 0.001), while job burnout positively predicted turnover intention (*β* = 0.16, *SE* = 0.03, *p* < 0.001). The indirect effect of job demands on turnover intention via job burnout was found to be significant (indirect effect *=* 0.08, 95% CI [0.048, 0.116]), thus supporting Hypothesis 2. Generally, this model accounted for 34.61% of the total effect.

### 3.5. Moderated Mediation Analysis

**Figure 2:**
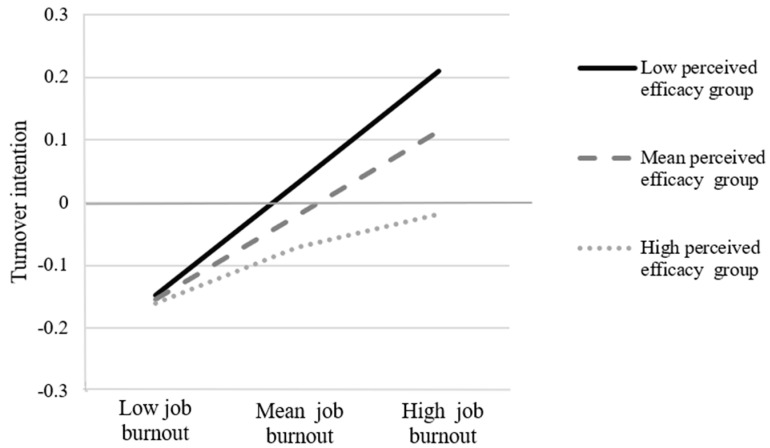
Moderating effect of perceived efficacy in overcoming the COVID-19 pandemic on the correlation between job burnout and turnover intention. Notes: “Low perceived efficacy group” means a low level of perceived efficacy in overcoming COVID-19; “Mean perceived efficacy group” means a mean level of perceived efficacy in overcoming COVID-19; and “high perceived efficacy group” means a high level of perceived efficacy in overcoming COVID-19.

Model 14 of the PROCESS macro was used to assess whether a perceived efficacy in overcoming the COVID-19 pandemic moderated the mediating effect of job burnout between job demands and turnover intention. In all analyses, we controlled for covariates, including gender, age, marital status, education, and income. As shown in Table 4, job demands positively predicted turnover intention (*β* = 0.15, *SE* = 0.03, *p* < 0.001). The association between burnout and turnover intention was found to be moderated by the perceived efficacy in overcoming the COVID-19 pandemic (*β* = −0.05, *SE* = 0.02, *p* = 0.048). The simple slope test in Figure 2 indicated that for the low level of perceived efficacy group (1 SD below the mean), job burnout had a stronger negative impact on turnover intention (*β* = 0.18, *SE* = 0.04, *p* < 0.001) than that of the mean level (*β* = 0.13, *SE* = 0.03, *p* < 0.001) and the high level of perceived efficacy group (1 SD above the mean) (*β* = 0.09, *SE* = 0.04, *p* = 0.027). That is, as job burnout increased, turnover intention increased more in the lower-perceived efficacy group and less in the higher-perceived efficacy group, respectively.

As indicated in Table 5, the indirect association between job demands and turnover intention was found to be significantly stronger for the prison officers with a low level of perceived efficacy (Indirect effect = 0.09, 95% CI [0.055, 0.131]). Meanwhile, this indirect relationship was also found to be significant but weaker for prison officers with a high level of perceived efficacy (Indirect effect = 0.04, 95% CI [0.006, 0.089]).

Table 6 shows that the index of moderated mediation’s bias-corrected 95% confidence interval did not include 0, and varied from −0.047 to −0.007, respectively. This indicated that the indirect effect of job demands on turnover intention via job burnout was moderated by the perceived efficacy in overcoming the COVID-19 pandemic, which supported Hypothesis 3.

## 4. Discussion

This study effectively elucidated the mechanism linking job demands and turnover intention among prison officers during the COVID-19 pandemic. The findings indicated that job demands had a positive impact on turnover intention, and this effect was mediated by job burnout. Furthermore, perceived efficacy in overcoming the COVID-19 pandemic moderated the relationship between job burnout and turnover intention. These findings supported the three hypotheses mentioned above, which will be discussed individually and in-depth later.

As COVID-19 can recur periodically and spreads swiftly, prison officers in China were facing tremendous pressure. This study is the first survey focused on Chinese prison officers’ occupational psychology during the COVID-19 pandemic. Its findings can serve as an effective reference and provide practical guidance for global prisons in addressing the issue of high turnover rates during future major public health incidents. Theoretically, this research expanded the applicability of the job demands–resources (JD–R) theory and the Hom-Griffeth’s employee turnover model. Furthermore, it elucidated the boundary effects of the mediation model in the specific context of COVID-19 by introducing the perceived efficacy in overcoming the COVID-19 pandemic as a moderating variable. These contributions further enhance our understanding of the underlying mechanisms involved in the relationships of job demands, burnout, perceived efficacy in overcoming the COVID-19 pandemic, and turnover intention during public health crises.

### 4.1. Job Demands and Turnover Intention

This study showed that job demands were positively associated with turnover intention among prison officers during the COVID-19 pandemic, which supported the first Hypothesis (H1), and accorded with previous research across various work contexts and employee populations [8,18,52]. Additional job demands, such as temperature checks and nucleic acid tests for prisoners have further intensified the workload and negative emotions experienced by the prison officers [53]. According to the Hom-Griffeth‘s employee turnover model, when prison officers’ work demands exceed their capacity, they would be dejected and feel it difficult to complete their tasks, resulting in an increased turnover intention [22,23]. Thus, the COVID-19 pandemic made prison officers work harder than before, placing more pressure on them. This increased prison officers’ anxiety and insecurity about their jobs, which led to their growing turnover intention day by day. Not only did they need to protect themselves from infection, but they also strived to prevent cross-infection among the prisoners. Thus, during the COVID-19 pandemic, prison officers’ high job demands easily led to an increased turnover intention.

### 4.2. The Mediating Role of Job Burnout

As expected, our results supported the second Hypothesis (H2), indicating that job burnout partially mediated the relationship between work demands and turnover intention among prison officers. This result aligned with the Hom-Griffeth’s employee turnover model, which posited those individuals who experienced high job demands easily develop job burnout, ultimately triggering thoughts of leaving. Previous studies have indicated that the depletion of working resources was the main predictor of professional burnout, which was related to quitting intention [54].

It is crucial to focus on the overall mediation results and on each connection in the model. We discovered a positive association for the first stage (i.e., job demands → burnout), corresponding with previous studies on other occupations [55,56]. On the one hand, according to the JD–R theory [6], increased work demands consumed prison officers’ mental and physical resources. Consequently, it might result in a loss in vigor and energy. On the other hand, based on the job demand control theory formulated by Karasek [57], job demands, and work control are two vital job characteristics related to burnout in a work context. The latter is the capacity of a person to manage their work-related activities [58], which plays a buffering role in job burnout caused by high job demands. During the COVID-19 pandemic, prison officers faced increased job demands and had to obey orders, leading to a perceived loss in job control [53]. In this way, prison officers felt they were instrumentalized, and lacked self-worth, resulting in alienation and burnout [59].

For the second stage (i.e., burnout → turnover intention), we also uncovered a positive association consistent with previous studies [60]. It revealed that job burnout was a significant factor leading to turnover intention, which has been validated across different occupational groups [21]. The social exchange theory (SET) [61] suggests that employees develop a social exchange relationship with their organizations based on a reciprocal exchange of resources and benefits. When employees experience job burnout, they may perceive that their organizations are not able to provide the sufficient job resources they require, resulting in a decreased identification with the organization and an increased quit intention [62]. Han et al.’s research was consistent with this theory, which showed that burnout reduced organizational commitments and increased turnover intention [63].

### 4.3. The Moderating Role of Perceived Efficacy in Overcoming the COVID-19 Pandemic

This research revealed that the perceived efficacy in overcoming the COVID-19 pandemic moderated the influence of burnout on turnover intention. Additionally, prison officers with a low perceived efficacy were more likely to leave than those with a high perceived efficacy. Our findings confirmed the third Hypothesis (H3).

Under Bandura’s efficacy model [64], people have two expectations about their behavior in the future. One is the outcome expectation, which is the idea that their actions will or will not cause a particular outcome. Another is the efficacy expectation, which refers to one’s opinion of the capacity to conduct the necessary behavior [65]. From research conducted on the general population, Gabriele and Dai pointed out that perceived efficacy of overcoming the COVID-19 pandemic affected people’s attitudes towards the pandemic, with those possessing a high perceived efficacy being more confident about controlling and defeating the pandemic [26,28]. Similarly, prison officers with a high perceived efficacy considered that the COVID-19 epidemic would be overcome sooner or later, and that the preventative efforts implemented were effective. Overall, this means that they had both outcome and efficacy expectations in overcoming the COVID-19 pandemic. As a result, they would not have the desire to leave as they anticipate fewer job demands and lesser burnout in the future.

According to the conservation of resources theory (CRT) [66], people seek to preserve and protect their resources. If employees perceive that the government can take effective steps to manage the pandemic and protect their work resources, they may be more likely to be hopeful about the future [67], which will reduce their desire to leave. Thus, it implied that the turnover intention of prison officers with a high perceived efficacy was less affected by burnout than those with a low perceived efficacy. We can conclude that the perceived efficacy in overcoming the COVID-19 pandemic was a protective factor against the influence of burnout on turnover intention.

### 4.4. Practical Contributions

Although the prevention of the COVID-19 pandemic has become routine, difficulties may occur for prison officers in similar public health incidents in the future. The implications of this study will also be helpful during future pandemics. In light of the results, we offer the following suggestions:

First, since high job demands were associated with a high turnover intention, prisons should allocate tasks to prison officers reasonably, and try not to add too much extra work for them when facing public health incidents.

Second, job burnout mediated the link between work demands and turnover intention, meaning that prisons should take effective steps to reduce prison officers’ job burnout, such as offering psychological support. In this way, prison officers will have adequate psychological resources to adapt to their intense workload and job pressure, thereby reducing their burnout.

Third, more attention should be paid to improving prison officers’ perceived efficacy in overcoming the COVID-19 epidemic. For one thing, prisons should implement well-planned workplace protocols for pandemic prevention and control. Complete preparation will increase prison officers’ confidence to overcome the pandemic. Furthermore, it would be useful to enhance the publicity about successful major public health events prevention cases. Encouraged by these cases, prison officers may be more likely to believe that they can deal with pandemic outbreaks in prisons and that difficulties are only temporary.

To sum up, this study provided a new perspective on dealing with burnout and turnover intention due to major public health events. The suggestions above can be used to mitigate the negative effects of public health events on prison operations.

### 4.5. Limitations and Future Directions

There are several limitations in the current study. First, we used questionnaires and a cross-sectional research design, making it impossible to infer the causality of the variables. Future research should employ experimental and longitudinal methods to further investigate this moderated mediation model. Second, the results were based purely on self-report measures, which may be prone to bias. For example, prison officers may have worries about their turnover intention becoming known by prison leaders. Therefore, future studies should use different techniques, such as behavioral or implicit measures, to investigate the associations among these variables. Third, this research was conducted with a sample of Chinese prison officers who were quite homogenous in culture, despite the high sample size. Future research should expand on these results in other cultural contexts.

## 5. Conclusions

In conclusion, the current research discovered that: (1) during the COVID-19 pandemic, job demands positively predicted the turnover intention of prison officers; (2) job burnout mediated the link between job demands and turnover intention; (3) the perceived efficacy in overcoming the COVID-19 epidemic moderated the association between job burnout and turnover intention. That is, based on the situation of the growing job demands for prison officers throughout the COVID-19 pandemic, the present study found a way by improving the perceived efficacy in overcoming the COVID-19 epidemic to reduce prison officers’ turnover intention, which was an evidence-based practical solution for similar major public health incidents in the future. In addition, as the first investigation of the occupational psychology of prison officers during the COVID-19 pandemic, this study provided a solid empirical basis for occupational psychology research on prison officers in future public health events.

## Figures and Tables

**Table 1:** Demographic details of the included prison officers (*N* = 1316).

Variable	Characteristic	Frequency	Percentage (%)
Gender	Male	766	58.2
	Female	550	41.8
Age	20–29	185	14.1
	30–39	394	29.9
	40–49	276	21.0
	≥50	461	35.0
Marital Status	Single	158	12.0
	Married	1081	82.1
	Divorced	72	5.5
	Widowed	5	0.4
Education	High School	29	2.2
	College	247	18.8
	Bachelor	1023	77.7
	Master or above	17	1.3
Average AnnualIncome	≤¥ 50,000	47	3.6
	¥ 50,001–¥ 100,000	398	30.2
	¥ 100,001–¥ 150,000	657	49.9
	¥ 150,001–¥ 200,000	197	15.0
	>¥ 200,000	17	1.3

**Table 2:** Descriptive and correlational statistics (*N* = 1316).

	M	SD	1.	2.	3.	4.
1. Job demands	20.96	4.53	—			
2. Job burnout	41.08	15.07	0.53 **	—		
3. Turnover intention	8.61	3.01	0.32 **	0.28 **	—	
4. Perceived efficacy	23.12	4.68	−0.25 **	−0.39 **	−0.19 **	—

Note. ** *p* < 0.01. M: mean; and SD: standard deviation.

**Table 3:** The mediating effect of job burnout.

Outcome	Predictors	*β*	*p*-Value	LLCI	ULCI
Job burnout	Job demands	0.52	<0.001	0.474	0.570
	*R*^2^ = 0.29, *F* = 89.87, *p* < 0.001				
Turnover intention	Job demands	0.15	<0.001	0.093	0.214
	Job burnout	0.16	<0.001	0.097	0.214
	*R*^2^ = 0.18, *F* = 40.60, *p* < 0.001				

Note. LLCI: lower limit of the 95% confidence interval; and ULCI: upper limit of the 95% confidence interval.

**Table 4:** The moderated mediating effect of perceived efficacy in overcoming the COVID-19 pandemic.

Outcome	Predictors	*β*	*p*-Value	LLCI	ULCI
Job burnout	Job demands	0.52	<0.001	0.474	0.570
	*R*^2^ = 0.29, *F* = 89.87, *p* < 0.001				
Turnover intention	Job demands	0.15	<0.001	0.088	0.209
	Job burnout	0.13	<0.001	0.071	0.193
	Perceived efficacy	−0.05	0.086	−0.106	0.007
	Job burnout × Perceived efficacy	−0.05	0.048	−0.091	−0.004
	*R*^2^ = 0.18, *F* = 32.89, *p* < 0.001				

Abbreviations: LLCI: lower limit of the 95% confidence interval; and ULCI: upper limit of the 95% confidence interval.

**Table 5:** Conditional indirect effect of job demands on turnover intention through job burnout at different levels of perceived efficacy in overcoming the COVID-19 pandemic.

Perceived Efficacy	Indirect Effect	Boot *SE*	Boot LLCI	Boot ULCI
Mean − 1 SD	0.09	0.02	0.055	0.131
Mean	0.07	0.02	0.035	0.104
Mean + 1 SD	0.04	0.02	0.006	0.089

Notes: LLCI: lower limit of the 95% confidence interval; ULCI: upper limit of the 95% confidence interval. Mean − 1 SD: low level of perceived efficacy in overcoming COVID-19 group; Mean: mean level of perceived efficacy in overcoming COVID-19 group; Mean + 1 SD: high level of perceived efficacy in overcoming COVID-19 group.

**Table 6:** Index of moderated mediation.

	Index	Boot *SE*	Boot LLCI	Boot ULCI
Perceived efficacy	−0.02	0.01	−0.047	−0.007

## Data Availability

The dataset in this research can be obtained upon request.
